# Simulating the Long-Term Impacts of the COVID-19 Pandemic on the Sustainability of the Population-Economy-Environment Nexus

**DOI:** 10.1007/s41885-021-00094-3

**Published:** 2021-08-26

**Authors:** Miguel Poblete-Cazenave

**Affiliations:** International Institute for Applied Systems Analysis (IIASA), Schlossplatz 1, A-2361 Laxenburg, Austria

**Keywords:** Environmental modelling, Simulation-based estimation, Slow-fast dynamics, Economy-population-environment nexus

## Abstract

The COVID19 pandemic has created a massive shock, unexpectedly increasing mortality levels and generating economic recessions all around the world. In recent years, several efforts have been made to develop models that link the environment, population and the economy which may be used to estimate potential longer term effects of the pandemic. Unfortunately, many of the parameters used in these models lack appropriate empirical identification. In this study, first I estimate the parameters of “Wonderland”, a system dynamics model of the population-economy-environment nexus, and posteriorly, add external GDP and mortality shocks to the model. The estimated parameters are able to closely match world data, while future simulations point, on average and regardless of the COVID19 pandemic, to a world reaching dangerous environmental levels in the following decades, in line with consensus forecasts. On the other hand, the effects of the pandemic on the economy are highly uncertain and may last for several decades.

## Introduction

The COVID-19 Pandemic that has affected the world in 2020 has been a significant shock, not only in terms of mortality, but also in terms of economic growth. Although, due to several factors, there is still no consensus on how large is the impact on mortality numbers (Beaney et al. 2020; Kontis et al. 2020; Roser et al. 2020), its impact on the economy has been massive, with several countries having their worst recessions on record (IMF 2020; OECD 2020; World Bank 2020). The scale as well as the unexpected nature of the pandemic overshadowed what has been in recent years probably the most debated topic in terms of sustainability, namely, climate change due to human intervention. The behavioral changes that have happened worldwide, specially in terms of mobility (e.g. lockdowns, border closures, see Kurita and Managi (2020), Katafuchi et al. (2021), and Nakamura and Managi (2020)), led to some people to suggest positive impacts in the environment (Le Quéré et al. 2020; Liu et al. 2020), which could be translated in improvements to population health, mortality reduction, and increased economic output, due to reductions in air pollution (Pandey et al. 2021). However, given the slow nature of climate change, it is unlikely that this would have any significant impacts in the overall trends. If anything, studies have shown that, although there was an improvement in air quality during lockdowns, these effects were only valid in the short-term, and pollution levels went back to the trend rapidly after cities started reopening (IEA 2020). Nevertheless, besides these purely direct effects on air pollution, there are other potential effects that may arise in the longer term, as the unexpected shocks to the economy and the population can have cascading effects due to their interconnected nature.

Therefore, this study attempts to add some insights into the possible long-terms effects of the COVID-19 pandemic on the population-economy-environment nexus at a global level that can be caused by the observed increases in mortality and GDP drops. To do that I use Wonderland (Sanderson 1994), a highly stylized simulation model of the population-environment-economy nexus. This model presents two main advantages for this purpose: first, its parsimonious nature provides a simple way to analyze these interactions, without the needs of massive data or computational power. Second, and most important, it is one of the few, if not the only model, that explicitly includes mortality as a variable and driver, instead of using population aggregates. In this sense, it allows us to independently add external mortality and GDP shocks without intervening other variables of the model at a specific point in time (i.e. 2020 and 2021), and posteriorly, simulate the effect of these shocks into the future. Nevertheless, it is important to clearly acknowledge that Wonderland is a simplistic “toy model” and, therefore, these results should be taken carefully. However, in order to try to overcome this limitation, I start the study by using empirical data to estimate parameters of the Wonderland Model in order to match the observed global trends in the past years. For this, I follow two approaches, one which performs the estimation of the parameters taking each equation of the model separately (Non Linear Least Squares), and another which estimates the parameters of the system as a whole (Indirect Inference). To the best of my knowledge, this is the first attempt to fully econometrically estimate the parameters of a global scale model of the population-economy-environment nexus.

The estimation procedures are able to fit the observed data quite closely, although a few of the parameters estimates are not very accurate. Moreover, a series of simulations using these estimated values (without the recently observed COVID-19 shocks) point to a world surpassing sustainability limits in the upcoming decades, in line with the forecasts of the majority of the more complex models, which gives support to the suitability of the Wonderland model for the analysis.

Building on this, I externally add the COVID-19 pandemic to the model for the years 2020 and 2021 using excess death estimates from Kontis et al. (2020) to simulate the shock in terms of mortality, and scenarios of the effects of COVID-19 on GDP from IMF (2020) for the short-term effects in the economy. Then, I use the Wonderland model to simulate pathways from the year 2020 onwards to find that, while the consequences on the economy should be persistent and expected to last between 40 to 70 years, the effects in the environment can be considered to be largely negligible. Although there have been studies that look at the potential effects of the pandemic on the population (e.g. Banerjee et al. (2020), Ghisolfi et al. (2020), and Marois et al. (2020)), the economy (e.g. Baker et al. (2020), Fernandes (2020), and Maliszewska et al. (2020); Gharehgozli et al. (2020), Mandel and Veetil (2020), and Jena et al. (2021)) and the environment(e.g. Forster et al. (2020), Saadat et al. (2020), and Zambrano-Monserrate et al. (2020)) this is the first study (also to the best of my knowledge) that looks at the potential consequences in all these aspects at the same time. In addition, the current studies tend to focus on the short and, at most, medium terms effects of the pandemic, while here the purpose is to see how much the longer term trends are affected, looking to potential cascading effects and associated pitfalls in the upcoming decades.

This paper is structured as follows. In the following section, I present and briefly describe the model from Sanderson (1994). Then, in Section “Parameter Estimation and Model Fit”, I present the procedures and the results from the estimation. In Section “Future Scenarios and the Impact of COVID-19” I show the results of the simulations using the estimated parameters from Section “Parameter Estimation and Model Fit”. In the last section, I present the conclusions.

## Modeling the Population-Economy-Environment Nexus

### Literature Review

The interactions between population, the economy and the environment has been a widely studied topic, especially in the last half century, where the threat of global warming started being apparent. There have been various attempts to model these interactions under different methodological approaches. One noticeable example here is the World3 system dynamics model (Meadows et al. 1972), which almost singlehandedly started the debate on climate change by forecasting a gloomy future scenario based on the conditions that the researchers found at that time. The most dominant modeling approach to this problem has been the use of Integrated Assesment Models (IAMs), for example, the DICE model (Nordhaus 1992) by the recent Nobel Prize winner William Nordhaus. These models have been heavily attacked (e.g. Cole et al. (1973), Simon (1981), Antimiani et al. (2015), Pindyck (2013), and Pindyck (2017)), most importantly, because many of its parameters and functional form assumptions can be considered arbitrary. Whether this diminishes the value of these models remains a matter of discussion (Nordhaus 2014; Weyant 2017). Interestingly, in some cases it has been found that some models’ predictions have been quite accurate (as it has been the case, for example, for the World3 model, as shown in Turner (2008)), although, as in all models of large-scale systems where human agents are involved, the accuracy of a forecast may not necessarily be the best measure whether the model is an adequate representation of the system.

Among the models following the system dynamics approach we have the Wonderland Model, first developed by Sanderson (1994) to study the interactions in terms of sustainability between the economy, population and the environment. It is a much more parsimonious model than its main counterpart, the World3 model, and therefore, it allows a simpler and direct analysis of the parts involved. Nonetheless, since its inception, it was shown that the model is very sensible to its parameter values, creating even a small literature on its stability due to the slow-fast dynamics it presents (e.g. Milik et al. (1996), Gragnani et al. (1998), and Wegenkittl et al. (1997)).

One of the major criticisms of these models is that their parameters values are arbitrary or poorly estimated. Here, taking advantage of its limited size, I estimate parameters for the Wonderland model in order to, first, find model parameters that generate a data close to what has been observed in the world during the past years, and, second, to simulate where the world is headed according to the estimated parameters within the Wonderland model framework. I use two estimation techniques. First, I use Non-Linear Least Squares, which, although straightforward, does not fully take into account the interaction between the different equations of the model. Second, and to overcome this issue, I use a simulation based estimation technique, Indirect Inference (Gourieroux et al. 1993), which has been used in a variety of structural econometric models, but also recently, within a system dynamics framework (Hosseinichimeh et al. 2016), as it allows us to account the system as a whole.

Finally, it is important to reiterate that this study is not intended as an addition nor an improvement on the modeling literature. On the contrary, here I take the Wonderland model as it was originally designed, aware of its possibilities but, at the same time, accepting its limitations and flaws, only making small tweaks in order to make the estimation of its parameters possible (details in Section “Parameter Estimation and Model Fit”).

### Wonderland Model Description

The Wonderland model consists of eight main equations, representing different factors that affect worldwide sustainability.

### Economy:

1 $$ \begin{array}{@{}rcl@{}} I_{t+1}&=&I_{t}\left[1+\gamma-(\gamma+\eta)(1-NK_{t})^{\lambda} \right] \end{array} $$

2 $$ \begin{array}{@{}rcl@{}} NI_{t}&=&I_{t}-PC_{t} \end{array} $$

### Population:

3 $$ \begin{array}{@{}rcl@{}} BR_{t}&=&\beta_{1}\left[\beta_{2}-\frac{e^{\beta_{3} NI_{t}}}{1+e^{\beta_{3} NI_{t}}}\right] \end{array} $$

4 $$ \begin{array}{@{}rcl@{}} DR_{t}&=&\alpha_{1}\left[\alpha_{2}-\frac{e^{\alpha_{3} NI_{t}}}{1+e^{\alpha_{3} NI_{t}}}\right]\left\{1+\alpha_{4}(1-NK_{t})^{\theta}\right\} \end{array} $$

5 $$ \begin{array}{@{}rcl@{}} N_{t+1}&=&N_{t}\left[1+\frac{BR_{t}-DR_{t}}{1000}\right] \end{array} $$

### Environment:

6 $$ \begin{array}{@{}rcl@{}} PF_{t}&=N_{t}I_{t}T_{t}-\kappa\left( \frac{e^{\epsilon PC_{t}N_{t}}}{1+e^{\epsilon PC_{t}N_{t}}}\right) \end{array} $$

7 $$ \begin{array}{@{}rcl@{}} NK_{t+1}&=\frac{e^{\ln\left( \frac{NK_{t}}{1-NK_{t}}\right)+\delta(NK_{t})^{\rho}-\omega PF_{t}}}{1+e^{\ln\left( \frac{NK_{t}}{1-NK_{t}}\right)+\delta(NK_{t})^{\rho}-\omega PF_{t}}} \end{array} $$

### Environmental Policy:

8 $$ \begin{array}{@{}rcl@{}} PC_{t}=\phi(1-NK_{t})^{\mu} I_{T} \end{array} $$

where the variables of the model are:

- *I*_*t*_ is per capita output in year *t*
- *NK*_*t*_ is stock of natural capital in year *t*
- *NI*_*t*_ is net per capita output in year *t*
- *PC*_*t*_ is per capita pollution control expenditure in year *t*
- *BR*_*t*_ is crude birth rate
- *DR*_*t*_ is crude death rate
- *N*_*t*_ is population at year *t*
- *PF*_*t*_ is pollution flow in year *t*
- *T*_*t*_ is technology

Equation 1 links the economy with the environment, as next period’s GDP depends on both current GDP and the stock of natural capita. The variables *γ* and *η* represent the growth rate and shrink rate when the natural capital is at its maximum or minimum values, respectively. Equation 2 is an accounting equation: net GDP under this model framework is GDP minus expenditures on pollution control. Net GDP is linked to both birth and death rates in Eqs. 3 and 4. In particular, both birth and death rates decrease with increases in output. Death rates are additionally linked with the environment. As we can see in Eq. 4, decreases in natural capital increase mortality. In Eq. 6 we see that pollution increases with output and population. There is a multiplicative effect of technology. It is assumed that technology improves over time at a constant rate, and that this decreases the effects of GDP and population on the environment. Pollution control also has a moderating effect on the flow of pollution. Natural capital evolves depending on the previous level of natural capital and the flow of pollution, as can be seen in Eq. 7. Finally, the pollution control expenditure (equation 8) is positively correlated with output and negatively with the level of natural capital.

Milik et al. (1996) presents a continuous-time version of the model which is used to analyze its slow-fast dynamics, that is, how the variables of the system evolve at different velocities. It is shown that, even without the addition of external shocks, the system dynamics can present chaotic behavior, as the model’s reaction to pollution flows is non-linear. This has been posteriorly studied in particular within the complexity literature (Gröller et al. 1996; Herbert and Leeves 1998; Leeves and Herbert 2002; Kohring 2006). The instability of the model creates an additional challenge to the estimation, as the optimization process followed to find the unknown parameters can diverge towards sub-optimal parameter estimates, and therefore, decreasing their accuracy, as it will be shown in the following section.

### Parameter Estimation and Model Fit

Estimation of the model parameters was done using two estimation methods: Non Linear Least Squares (NLLS) and Indirect Inference on world aggregate data starting from 1970 to 2010. The dataset includes global GDP and population numbers to account for the economy and demographic aspects of the model. As a proxy of natural capital I use the total concentration of “Kyoto” greenhouse gases, while the worldwide emission of greenhouse gases is used as a proxy of pollution flow. Finally, I use the estimated RD&D budgets by region from the IEA as a proxy of pollution control expenditure. As in Sanderson (1994), GDP per capita, population and technology were normalized to have a value equal to 1 in 1970. Additionally, natural capital was normalized to have a value of 1 if the CO_2_ levels are of 300 ppm and to a value of 0 if the CO_2_ levels are of 500 ppm, and emissions data was normalized to have a starting value in line with Sanderson (1994). It is here important to notice that, given that the Wonderland model is intended as a simplification of a highly complex system, a clear causal identification of the parameters is not the main objective here, but to find estimates that can be help to use this model as a somewhat realistic, albeit simplified representation.

Probably the most straightforward method to estimate the unknown parameters of the Wonderland model is through a Non Linear Least Squares estimation of Eqs. 1 to 8. Since, as briefly discussed at the end of the previous section, Wonderland is a highly chaotic model with multiple crashing points, the Non Linear Least Squares algorithm was implemented using Simulated Annealing in order to avoid reaching non-global optimal parameters depending on the starting values for the estimation. I independently estimate the equations that include parameters that need estimating, namely, Eqs. 1, 3, 4, 6, 7 and 8. Joint estimation of the equations using Non Linear Least Squares under different specifications (i.e. different weighting methods) repeatedly provided, after several tries, a very poor fit to the data, and therefore, this option was disregarded.

As an alternative that could capture the whole dynamics of the model, I use Indirect Inference, a simulation based method of moments estimation were the objective is to simultaneously capture several features of the data, unlike Non Linear Least Squares, which treats each of the equations of the model as independent. Summarizing, the method starts by creating a simulated dataset using a initial guess of the unknown parameters of the model. Posteriorly, a series of auxiliary statistical models are calculated both in the empirical data and in the simulated dataset. Finally, using an optimization routine, it iterates on the parameters guesses of the model in order to minimize the distance between the auxiliary models in both the empirical and the simulated data.

I make two additions to the model, for estimation purposes. In Sanderson (1994), technology, a variable that combines the effects of new technologies in production and pollution control, is assumed to be the exogenous variable that follows a deterministic trend and, therefore, is not considered in the main equations of the model. Here, however, for estimation purposes and to reflect unexpected changes of technology that have happened in the world, I add an exogenous shock term *μ*_*t*_. Hence, technology follows:

9 $$ \begin{array}{@{}rcl@{}} T_{t+1}=\psi T_{t}+{\upsilon^{T}_{t}} \end{array} $$

with ${\upsilon _{t}^{T}}\sim \mathcal {N}(0,{\sigma _{1}^{2}})$. Similarly, I also add a GDP shock, transforming equation(1) into:

10 $$ \begin{array}{@{}rcl@{}} I_{t+1}=I_{t}\left[1+\gamma-(\gamma+\eta)(1-NK_{t})^{\lambda} \right]+{\upsilon^{I}_{t}} \end{array} $$

with ${\upsilon _{t}^{I}}\sim \mathcal {N}(0,{\sigma _{2}^{2}})$.

The following procedure was done for the estimation of the unknown parameters . First, to help with the Indirect Inference estimation, the values of the *β* s, *ϕ*, *μ* and *σ*_1_ were estimated separately using OLS and NLLS, respectively, as Eqs. 3, 8 and 9 are well-behaved enough to provide significant and consistent estimates without resorting to more complicated estimation techniques. Posteriorly, auxiliary models were chosen to reflect some characteristics of the Wonderland model, as well to reflect the characteristics of the unknown parameters in the real data. In particular, the following linear regressions where used as the first set of auxiliary models:

$$ \begin{array}{@{}rcl@{}} \ln(I_{t})&=&{\beta_{0}^{1}}+{\beta_{1}^{1}}\ln(I_{t-1})+{\beta_{2}^{1}}\ln(NK_{t}-1)+\varepsilon\\ \ln(DR_{t})&=&{\beta_{0}^{3}}+{\beta_{1}^{3}} \ln(I_{t})+{\beta_{2}^{3}}\ln(PC_{t})+{\beta_{3}^{3}}\ln(NK_{t})+\varepsilon\\ PF_{t}&=&{\beta_{0}^{5}}+{\beta_{1}^{5}} N_{t}I_{t}+{\beta_{2}^{5}} PC_{t}+\varepsilon\\ NK_{t+1}&=&{\beta_{0}^{6}}+{\beta_{1}^{6}} NK_{t}+{\beta_{2}^{6}} PF_{t}+\varepsilon \end{array} $$

in order to capture the features of equations (10), (4), (6) and (7) in the data. Additionally, to capture the features of the remaining time series, the following autoregressive models were included as the second set of auxiliary models:

$$ \begin{array}{@{}rcl@{}} I_{t}&=&{\phi_{1}^{1}}+{\phi_{2}^{1}}I_{t-1}+{\phi_{3}^{1}}I_{t-2}+\varepsilon_{t}\\ NK_{t}&=&{\phi^{2}_{1}}+{\phi_{2}^{2}}NK_{t-1}+\varepsilon_{t}\\ N_{t}&=&{\phi_{1}^{3}}+{\phi_{2}^{3}}N_{t-1}+{\phi_{3}^{3}}N_{t-2}+{\phi_{4}^{3}}N_{t-3}+\varepsilon_{t}\\ DR_{t}&=&{\phi_{1}^{5}}+{\phi_{2}^{5}}DR_{t-1}+\varepsilon_{t}\\ PF_{t}&=&{\phi_{1}^{7}}+{\phi_{2}^{7}}PF_{t-1}+{\phi_{3}^{7}}PF_{t-2}+{\phi_{4}^{7}}PF_{t-3}+\varepsilon_{t} \end{array} $$

Finally, five moments containing the mean values of the the series from the equations not previously estimated were also added as the final set of auxiliary models.

**Fig. 1:**
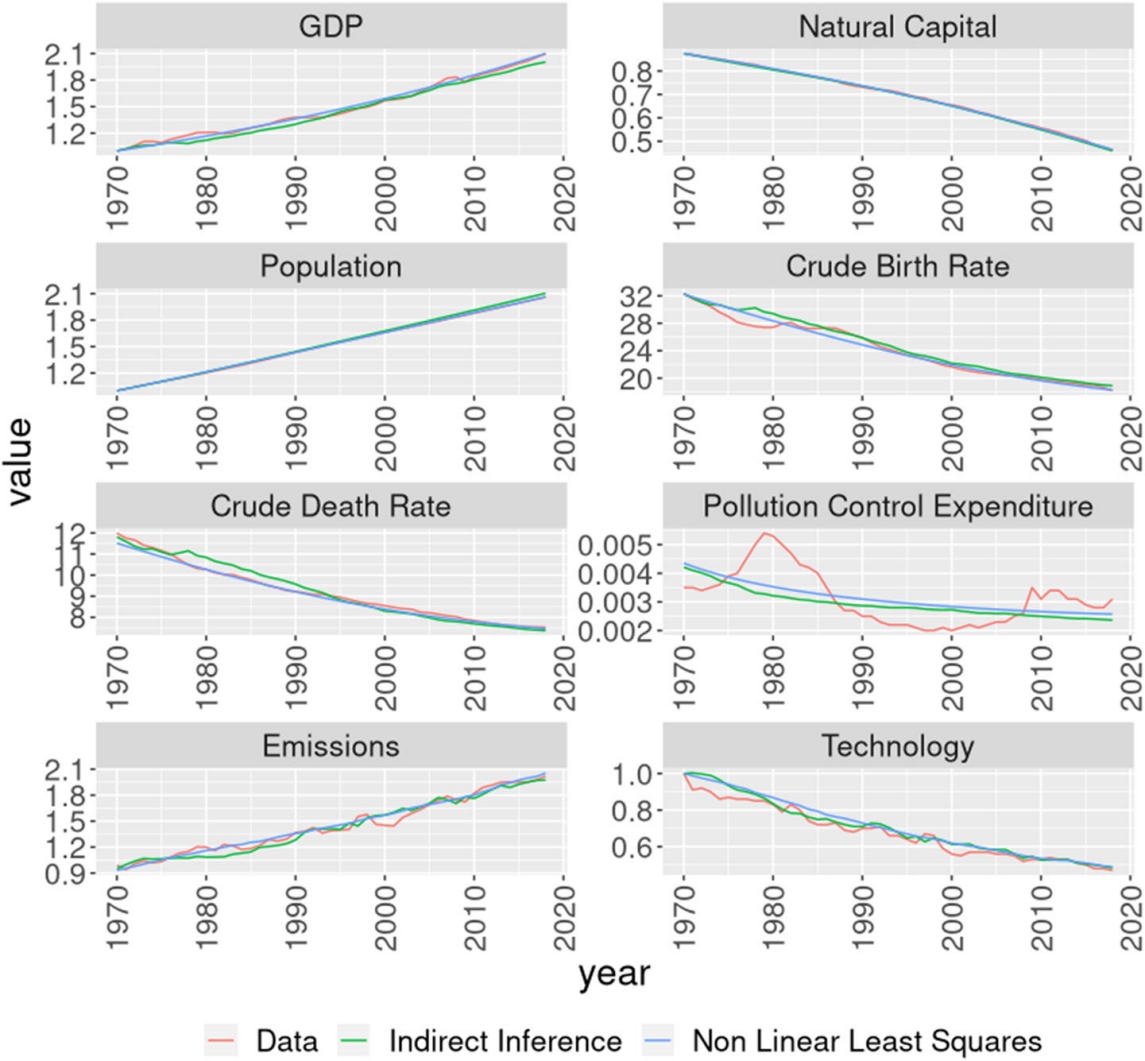
Fit of the estimation models

The estimated parameter values can be observed in Table 1. First, it is noticeable that the estimated values are vastly different from the parameters in Sanderson (1994). However, this should not come as a surprise, as those parameters where selected for illustration purposes and did not have references to real data. Additionally, some parameters are not significant^1^, especially in the NLLS estimation. This is also not surprising mainly because the data consists of series dating back up to 1970. In such a short period it is hard to be able to pin down parameters related to the relation between the economy and the environment, especially considering that nothing catastrophic has yet occurred^2^. This explains also why the Indirect Inference estimates are more significant, as the inclusion of other related variables in the model help identifying some of the parameters. Nevertheless, the model fit that can be seen graphically in Fig. 1 show that the estimated parameters are able to generate a simulated data that follows quite closely the real observed data.

**Table 1:** Parameter: Estimated Values using NLLS, Indirect Inference and Values from Sanderson (1994)

Parameter	Sanderson (1994)	NLLS		Indirect Inference	
*γ*	0.04	0.01559	(***)	0.02188	(***)
*η*	0.04	5.05100		0.00000	
*λ*	2	59.32897	(*)	0.96732	(*)
*β*1	35	116.73820	(***)	118.48760	(***)
*β*2	1.5	1.13608	(***)	1.13286	(***)
*β*3	0.08	1.82388	(***)	1.82711	(***)
*α*1	20	44.85680	(***)	0.70002	(***)
*α*2	1.5	1.148700	(***)	1.13962	(***)
*α*3	0.09	2.27164	(***)	1.88030	(***)
*α*4	29	0.06487	(*)	61.52557	(*)
*𝜃*	15	0.03770	(*)	0.00000	(***)
*ψ*	0.99	0.98552	(***)	0.98241	(***)
*κ*	0.02	0.16218	(***)	0.05293	(***)
*𝜖*	1	12.35102	(*)	530.15737	(***)
*δ*	0.2	0.02995		0.34411	(***)
*ρ*	0.1	0.58422	(*)	0.00533	(*)
*ω*	0.5	0.02607	(**)	0.17232	(***)
*ν*	1	0.99208	(***)	0.96444	(***)
*ϕ*	2	0.00071	(***)	0.00069	(***)
*μ*	0.99	-0.87220	(***)	-0.86965	(***)
*σ*_1_		0.00244	(***)	0.01231	(***)
*σ*_2_				0.00540	(***)

## Future Scenarios and the Impact of COVID-19

Using the parameters estimated in the previous section, we can simulate future scenarios. I propose four scenarios: one counterfactual where the COVID19 pandemic never happened, and three COVID19 scenarios (baseline, downside and upside). To analyze these potential futures in terms of sustainability, Sanderson (1994) provides two criteria: first, that net per capita income should not fall by 30 percent or more from its peak level, and second, that the crude death rate should not rise by 50 percent or more from its lowest level. Here, I will add another criterion regarding the environment: CO_2_ levels should not surpass the 450 ppm.

Given that, as stated in the introduction, it has been found that the direct effects on air quality of the lockdowns is negligible, the COVID19 scenarios are done taking into consideration only GDP and mortality shocks. Two sources were used as input in the creation of these scenarios. First, for the GDP shocks, I take as an input the latest scenarios from the International Monetary Fund (IMF 2020). For excess mortality, I use input from Kontis et al. (2020). Three scenarios are designed: a baseline scenario, using the baseline GDP scenario of IMF (2020) and the mean excess mortality observed in Kontis et al. (2020); a downside scenario, using the downside scenario of IMF (2020) and the upper bound of the confidence interval of Kontis et al. (2020); and an upside scenario, with the upside scenario of (IMF 2020) and the lower bound of the confidence interval of Kontis et al. (2020). Additionally, given that mortality is only given for 2020, it is assumed that the rate is halved in 2021. These values can be found in Table 2.

**Table 2:** COVID scenario assumptions

	Year	Baseline	Downside	Upside
GDP Growth*	2020	-4.40%	-5.15%	-4.40%
	2021	5.20%	2.34%	5.65%
Excess Mortality**	2020	23.0%	38.0%	7.0%
	2021	11.5%	19.0%	3.5%

^*^ Values from IMF (2020)

^**^ Values for 2020 from Kontis et al. (2020), 2021 assumed to be half of 2020

**Fig. 2:**
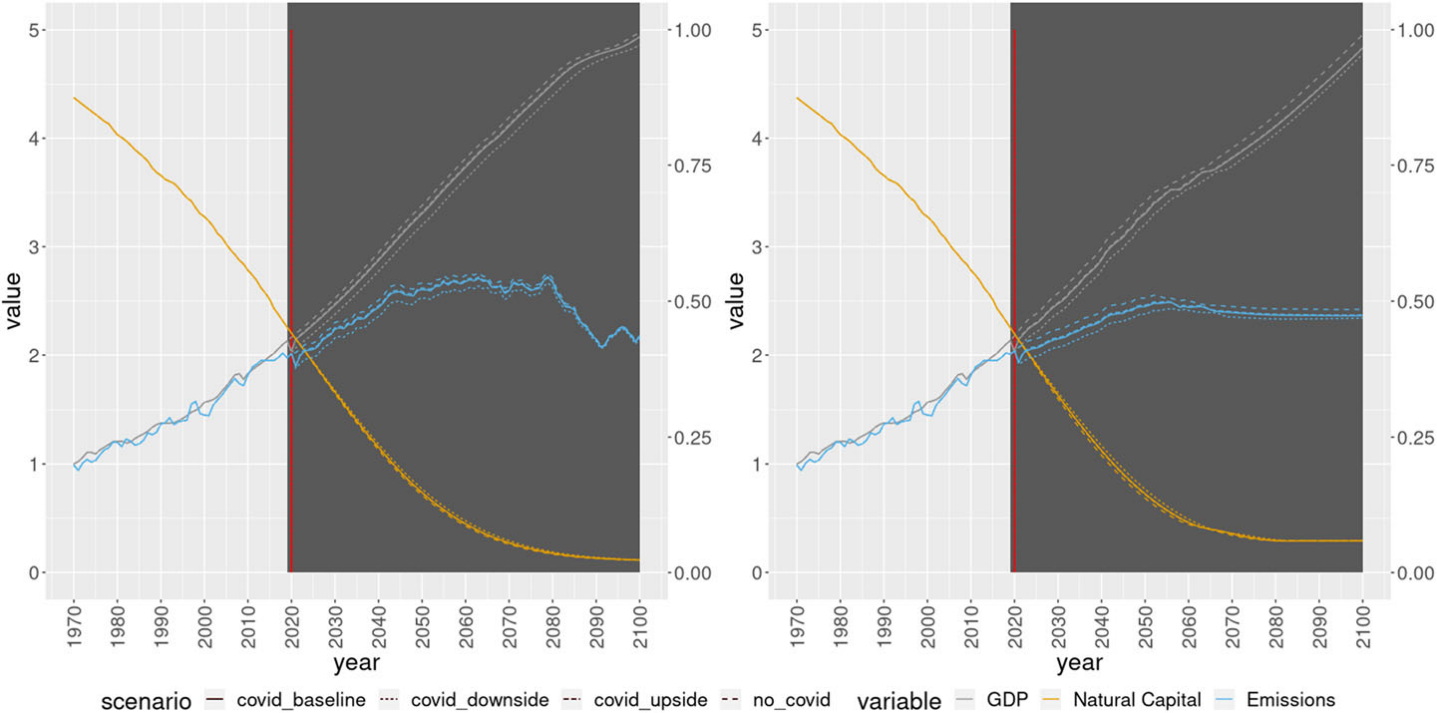
World GDP (left axis), pollution flow (left axis) and natural capital (right axis), non linear least squares (left panel) and indirect inference (right panel)

**Fig. 3:**
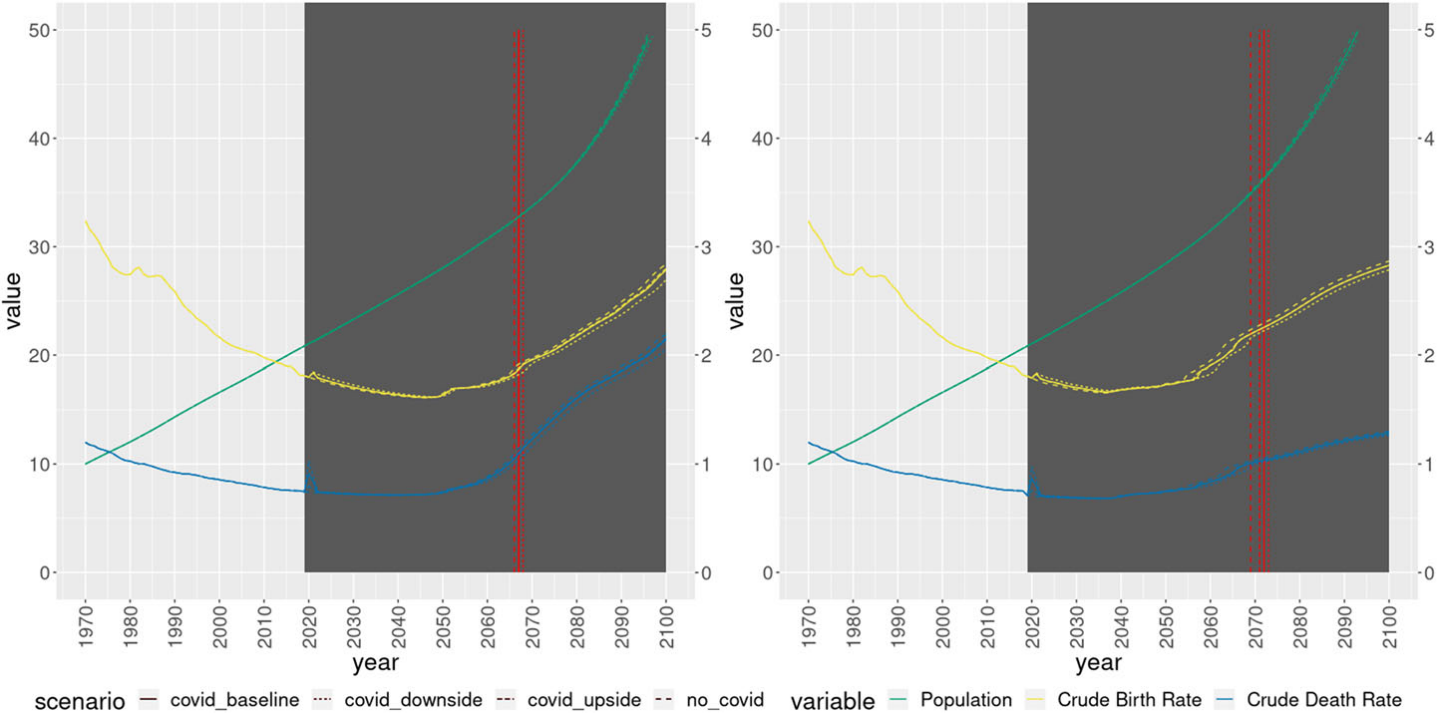
World population (right axis), crude birth rate (left axis) and crude death rate (left axis), non linear least squares (left panel) and indirect inference (right panel)

**Fig. 4:**
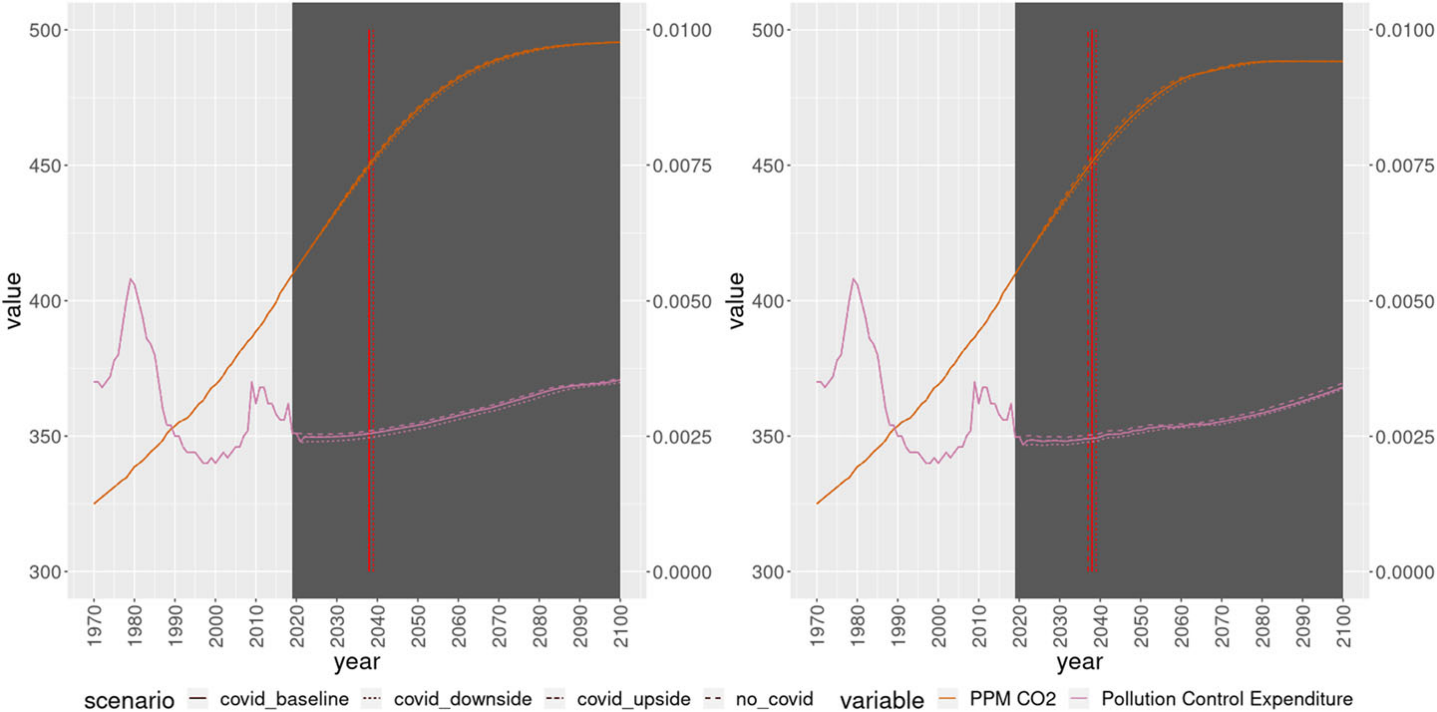
World levels of CO2 (left axis) and pollution control expenditures (right axis), non linear least squares (left panel) and indirect inference (right panel)

A total of 100 bootstrap simulations were performed. The average result of the simulations can be seen in Figs. 2, 3, 4. First, it is reassuring to see that the simulations using Indirect Inference do not provide much different results than the simulations using Non Linear Least Squares. Moreover, within the scenarios, there are no massive differences. If anything, the largest differences within scenarios, under both set of parameter estimates, can be seen in terms of GDP, emissions (Fig. 2) and crude birth and death rates (Fig. 3). In particular, the baseline and the upside scenario remain quite close, with the No COVID19 scenario and the downside scenario above and below them, respectively.

**Fig. 5:**
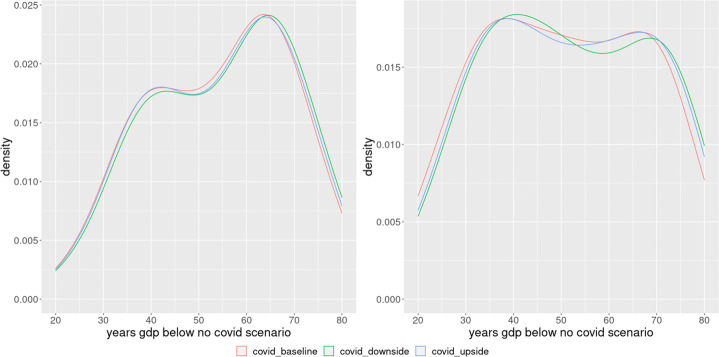
Kernel density estimate of years with a GDP below the No COVID19 Scenarios, non linear least squares (left panel) and indirect inference (right panel)

The differences that arise from COVID seem to be persistent over time. For example, in terms of GDP, on average, there seems to be a convergence only by the end of the century (Fig. 2). However, there is a wide distribution among the different bootstrap scenarios. As we can see in Fig. 5 which shows the distribution of the number of years in each COVID19 scenario where the GDP was below the GDP of the No COVID19 scenario, most of the scenarios clustering between 40 to 70 years of persistence, with a minimum of around 20 years of divergence.

As per the sustainability criteria, there are no big differences between the different sets of estimates. In particular, as we can see in Table 3, on average, there is no expected economic crash beyond the external shock of the COVID crash, the timing of the population crashes differ by around 3 years for the different set of estimates, while there are no major differences in the timing of the environmental crash. COVID19 doesn’t create much difference for the environment, it just delays the environmental crash by at most one year if we compare the No COVID19 scenarios with the baseline and upside scenarios, and by at most two years in the case of the downside scenario. However, there are some delays in the population crashes under the Indirect Inference estimates (btw. 2 to 4 years depending on the scenario).

**Table 3:** Crash years in different scenarios

		Scenario	Crash Years
Economy	Non Linear Least Squares	No COVID19	-
		COVID19 Baseline	2020
		COVID19 Downside	2020
		COVID19 Upside	2020
	Indirect Inference	No COVID19	-
		COVID19 Baseline	2020
		COVID19 Downside	2020
		COVID19 Upside	2020
Population	Non Linear Least Squares	No COVID19	2066
		COVID19 Baseline	2067
		COVID19 Downside	2068
		COVID19 Upside	2067
	Indirect Inference	No COVID19	2069
		COVID19 Baseline	2072
		COVID19 Downside	2073
		COVID19 Upside	2071
Environment	Non Linear Least Squares	No COVID19	2038
		COVID19 Baseline	2038
		COVID19 Downside	2039
		COVID19 Upside	2038
	Indirect Inference	No COVID19	2037
		COVID19 Baseline	2038
		COVID19 Downside	2039
		COVID19 Upside	2038

**Fig. 6:**
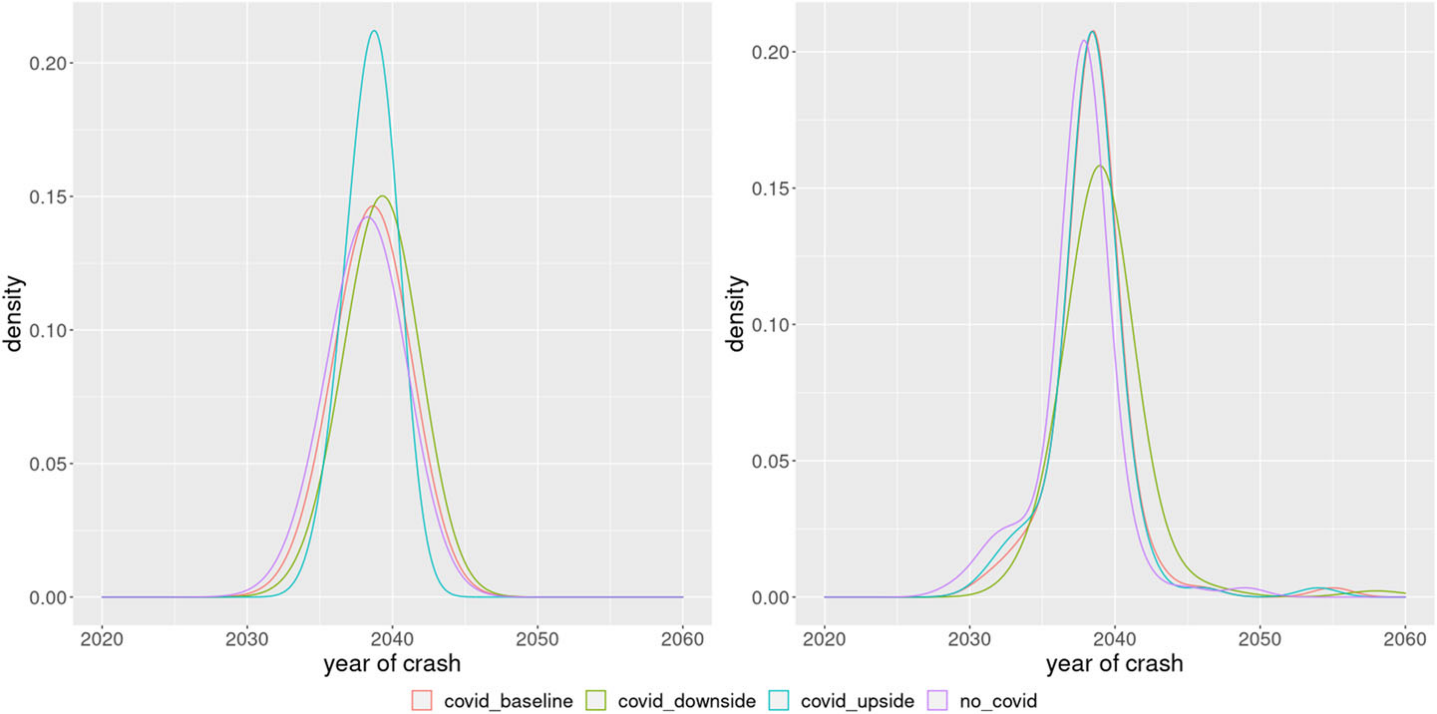
Distribution of the environmental crash: kernel density estimate, non linear least squares (left panel) and indirect inference (right panel)

The most interesting takeout nevertheless is in the case of the environmental sustainability. Per our criteria, under both set of estimates and on all scenarios, the world is expected to fall below the sustainability threshold around the years 2037$\sim $2039. These estimates are much more robust in terms of distribution, as we can see graphically in Fig. 6 and numerically in Table 4.

**Table 4:** Distribution of the environmental crash: average and confidence interval

	Non-linear least squares		Indirect inference	
	Mean	Std. Dev.	Mean	Std. Dev.
COVID19 Baseline	2038.6	1.330	2038.4	2.767
COVID19 Downside	2039.2	1.299	2039.2	2.736
COVID19 Upside	2038.5	1.324	2038.3	2.745
No COVID19	2038.1	1.374	2037.5	2.686

## Conclusions and Discussion

This work is intended as a first approximation to the potential long-terms effects of the COVID pandemic. Indeed, Wonderland is not a full-fledge Integrated Assessment Model, however, its ability to replicate real trends with our estimated parameters, as well as its correspondence with other such models in terms of the trajectories of PPM CO2 emissions, gives support to the results of this study. In this sense, given current trends, there is little room to improve the climate situation in the medium-term and we will see the world reaching dangerous levels in terms of sustainability of the environment by the end of the next decade. This can result in population crises by the second half of the century. However, no climate-induced economic crises seem to be in the horizon.

This can be seen as a reassuring result, nevertheless, there are some reasonable reasons to be uncertain about this takeout in particular. The first is about data. Since the worse effects of climate change are still expected in the future, past data may not be insightful enough to attempt a credible inference in this respect. Closely related to that, and something that this study does not attempt, is that the functional forms assumed may not be appropriate to truly capture the interactions between the relevant agents. Indeed, Wonderland is a highly simplified “toy model” that reduces the interactions among eight equations, and therefore, it cannot be expected to be a realistic representation of reality. Nevertheless, this is a short-coming that is shared among all models, as certainly no one can tell how exactly the economy may react to an environmental crisis. This is also evident from the potential COVID19 effects on GDP, which, unlike the climate effects, they seem to be highly uncertain in terms of their duration, with a range spanning several decades of difference.

Still, even disregarding the magnitude of the uncertainty, it is clear that there will be long-term effects in the economy. And, although here Wonderland is used as a global model and does not make distinctions between countries, it is most likely that the persistence of these effects will be driven by countries whose capacity to reactivate their economy is much more limited. Moreover, if the projections regarding vaccination rates in different countries are accurate (Duke Global Health Innovation Center 2021), those countries with already limited economic capacity to cope may be pushed to continue enforcing lockdown policies, adding up to the damaging effects in their economy. Therefore, unless global level policies are in place to support vaccination efforts, the outlook for these countries remains grim, pushing the expected recovery times of the global economy to the upper end of the presented distribution.

Finally, it is important to reiterate that, interestingly, the estimated model forecasts similar scenarios as the majority of the more complex and less tractable calibrated Integrated Assessment Models, namely, that, if nothing changes significantly in terms of policy, the world should go over the 450ppm limit within the next two decades. Moreso, even a worldwide crisis that forced massive behavioral changes in the population doesn’t seem to make any difference. In terms of policy, this seems to show that even a worldwide reduction of individual transport related emissions would not be enough to prevent an environmental catastrophe, unless there are also large structural changes in the production side. As stated before, there is still much need for better data to get more robust results. Unfortunately, the only way to obtain better data is to wait until the world starts suffering from the consequences of the environmental change, and therefore, it will be already too late to do something about it. Can we afford the wait?

## Data Availability

The datasets generated during and/or analysed during the current study are available from the corresponding author on reasonable request.

## Footnotes

1 The significance of the parameters was estimated using 1000 bootstrapping iterations.

2 In this sense, data for previous years, although possible to obtain, would not improve the estimation, as the consequences of climate change started being more visible in the current millennium.
